# Ectoine, from a Natural Bacteria Protectant to a New Treatment of Dry Eye Disease

**DOI:** 10.3390/pharmaceutics16020236

**Published:** 2024-02-05

**Authors:** Xin Chen, Na Lin, Jin-Miao Li, Haixia Liu, Anmar Abu-Romman, Ebru Yaman, Fang Bian, Cintia S. de Paiva, Stephen C. Pflugfelder, De-Quan Li

**Affiliations:** 1 Ocular Surface Center, Cullen Eye Institute, Department of Ophthalmology, Baylor College of Medicine, Houston, TX 77030, USA; xin.chen@bcm.edu (X.C.); na.lin@bcm.edu (N.L.); jinmiaoli@hotmail.com (J.-M.L.); anrmjo9@gmail.com (A.A.-R.); ebru.yaman@bcm.edu (E.Y.); fangbian05@gmail.com (F.B.); cintiadp@bcm.edu (C.S.d.P.); stevenp@bcm.edu (S.C.P.); 2 National Clinical Research Center for Ocular Diseases, Eye Hospital, Wenzhou Medical University, Wenzhou 325027, China; 3 State Key Laboratory of Ophthalmology, Zhongshan Ophthalmic Center, Guangdong Provincial Key Laboratory of Ophthalmology and Visual Science, Sun Yat-Sen University, Guangzhou 510060, China; 4 Allergan, an AbbVie Company, Irvine, CA 92612, USA; haixia.liu16@abbvie.com

**Keywords:** cornea, dry eye, ectoine, inflammation, murine model

## Abstract

Ectoine, a novel natural osmoprotectant, protects bacteria living in extreme environments. This study aimed to explore the therapeutic effect of ectoine for dry eye disease. An experimental dry eye model was created in C57BL/6 mice exposed to desiccating stress (DS) with untreated mice as controls (UT). DS mice were dosed topically with 0.5–2.0% of ectoine or a vehicle control. Corneal epithelial defects were detected via corneal smoothness and Oregon Green dextran (OGD) fluorescent staining. Pro-inflammatory cytokines and chemokines were evaluated using RT-qPCR and immunofluorescent staining. Compared with UT mice, corneal epithelial defects were observed as corneal smoothness irregularities and strong punctate OGD fluorescent staining in DS mice with vehicle. Ectoine treatment protected DS mice from corneal damage in a concentration-dependent manner, and ectoine at 1.0 and 2.0% significantly restored the corneal smoothness and reduced OGD staining to near normal levels. Expression of pro-inflammatory cytokines (TNF-α, IL-1β, and IL-6) and chemokines CCL3 and CXCL11 was significantly elevated in the corneas and conjunctivas of DS mice, whereas 1.0 and 2.0% ectoine suppressed these inflammatory mediators to near normal levels. Our findings demonstrate that ectoine can significantly reduce the hallmark pathologies associated with dry eye and may be a promising candidate for treating human disease.

## 1. Introduction

Dry eye is a common ocular inflammatory disease with increasing prevalence worldwide [1,2]. Patients with dry eye often suffer ocular discomfort, irritation, and visual disturbance, which reduce the quality of life [3]. Current dry eye therapies include tear supplementation, tear volume stimulation, biological tear substitutes, anti-inflammatory therapy, and essential fatty acids, which relieve dry eye symptoms [4,5] but may not prevent disease progression. It is important to develop a new line of treatment targeting the major etiological and pathological factors that cause dry eye.

Dry eye disease has been defined as a multifactorial disease of the ocular surface [6]. Among a variety of etiological and pathological factors, the tear hyperosmolarity has been recognized as one of the core etiological mechanisms [7] that induce ocular inflammation [8,9] in dry eye diseases. Topical treatment with osmoprotectants was recently developed by specifically targeting hyperosmolarity, a major etiological factor in dry eye disease [10]. Osmoprotectants are small molecules and compatible solutes that can restore isotonic cell volume and stabilize protein function without perturbing cellular macromolecules, which may protect cells by enhancing their adaptation to hyperosmolarity [11,12].

Ectoine (1,4,5,6-tetrahydro-2-methyl-4-pyrimidinecarboxylic acid) is a compatible water molecule-binding solute, and a natural osmoprotectant produced by several bacterial species that survive well in extreme conditions of salinity, drought, irradiation, pH and temperature [13]. Ectoine is a strong cell protectant with a unique effect of preferential hydration [14]. Through its reorganization of water molecules, ectoine works to exclude osmolytes from interacting with proteins and membranes, which stabilizes their native structure and morphology and protects cells from environmental stress [15].

Ectoine was also found to inhibit the inflammatory responses in respiratory and skin cells and has been used in some chronic inflammatory diseases [16]. For example, the topical application of ectoine has been used to treat rhinitis sicca and acute pharyngitis [17], as well as on skin lesions to relieve symptoms of atopic dermatitis [18]. For ocular disease, ectoine has recently been used in clinical trials to treat seasonal allergic rhinoconjunctivitis [19,20]. However, ectoine’s ability to protect against the inflammation associated with dry eye disease and its potential mechanism remain poorly understood.

Our team hypothesized that the strong osmoprotective effects of ectoine on bacteria could have a potential therapeutic application for the treatment of human dry eye disease. To test this, we recently investigated the novel role of ectoine in enhancing corneal epithelial cell viability and barrier function in an in vitro hyperosmotic induced dry eye model using primary human corneal epithelial cells. The study also showed that ectoine achieved the protective effect by promoting the anti-inflammatory cytokine IL-37, which suppressed pro-inflammatory cytokines and cathepsin S induced by hyperosmotic stress. In the present study, we further explored the potential therapeutic effect of ectoine on ocular surface cell protection and anti-inflammation in dry eye disease using a murine model exposed to desiccating stress.

## 2. Materials and Methods

### 2.1. Animal

C57BL/6J mice were purchased from the Jackson Laboratories (Bar Harbor, ME, USA) and housed in specific pathogen-free conditions in microisolator cages at the facilities of Baylor College of Medicine. Mice were kept on diurnal cycles of 12 h/light and 12 h/dark with ad libitum access to food, water, and environmental enrichment.

The animal research protocol was approved by the Institutional Animal Care and Use Committee (IACUC), Center for Comparative Medicine, Baylor College of Medicine (AN-2032), and it conformed to the standards in the ARVO Statement for the Use of Animals in Ophthalmic and Vision Research and the NIH Guide for the Care and Use of Laboratory Animals [21].

### 2.2. Mouse Model of Experimental Dry Eye Disease 

An experimental dry eye model was induced via desiccating stress (DS) in female C57BL/6 mice (DS mice) using our previously published methods [22,23]. In brief, mice aged 8–10 weeks were exposed to an air draft and 20–30% low ambient humidity in the controlled environment of Darwin Chambers with subcutaneous injection of 0.5 mg/0.2 mL of scopolamine hydrobromide (Sigma-Aldrich, St. Louis, MO, USA) four times a day (8 AM, 11 AM, 2 PM, and 5 PM) for 5 consecutive days.

### 2.3. Ectoine Topical Treatment

The DS mice were treated with topical administration via instillation of 5 µL/eye of ectoine (provided by AbbVie, Inc., North Chicago, IL, USA) in phosphate buffered saline (PBS) at different concentrations (0.5, 1.0 and 2.0%), or PBS vehicle, 4 times daily during exposure to desiccation for 5 days. The untreated age- and gender-matched control mice (UT) were maintained in a normal environment at 50–75% humidity. On day 5, clinical signs of dry eye and corneal damage were assessed via a stereo microscopy and Oregon Green dextran fluorescein staining. To evaluate ocular inflammatory markers, whole eyeballs, corneal epithelia, and conjunctival tissues were collected after animal euthanasia. Six mice per group were used for each experiment, and each experiment was repeated at least three times.

### 2.4. Evaluation of Corneal Smoothness 

With the approach previously reported [23,24], corneal smoothness was assessed in 10 eyes of each group of mice: UT, DS + PBS, and DS + Ectoine at three concentrations (0.5, 1.0, and 2.0%), from three different sets of experiments. Images of a reflected white ring from the fiberoptic ring illuminator of the stereoscopic zoom microscope (SMZ 1500; Nikon, Tokyo, Japan) were taken immediately after euthanasia, which occurred 2 h after instillation of the last treatment drops. The ring light was firmly attached and surrounded the bottom of the microscope objective. The projected ring light was reflected off a wet surface, so the ring regularity was dependent on the smoothness of the corneal surface. The projected ring was divided into four quarters, each quarter being 3 clock h. The corneal irregularity was scored based on the number of distorted quarters in the reflective ring using a 5-point scale: 0, no distortion; 1, distortion in one quarter; 2, distortion in two quarters; 3, distortion in three quarters; 4, distortion in all four quarters; and 5, severe distortion in which no rings could be identified. Two masked observers rated the scores on the digital images.

### 2.5. Evaluation of Corneal Defect via Oregon Green Dextran (OGD) Fluorescent Staining

On the afternoon of the fifth day, corneal defect and permeability was assessed by using OGD, which is a conjugated fluorescent dye 488 of a 70 kDa molecular size (Thermo Fisher Scientific, Eugene, OR, USA) as described previously [23,24]. The procedure consisted of 1.0 µL of 50 mg/mL OGD instilled into the eyes for one minute, followed by euthanasia with isoflurane inhalation and cervical dislocation. Eyes were then rinsed with 1 mL of balanced salt solution (BSS) twice. Excess liquid was blotted with filter papers without touching the cornea. Digital pictures on both eyes of each animal were taken with an exposure time of 200 ms for evaluation using a Nikon SMZ1500 stereo microscope at 470 nm excitation and 488 nm emission wavelengths. The mean fluorescence intensity in the central cornea with a diameter of 2 mm was evaluated from digital images using NIS Elements (version 3.0) software (Nikon USA, Melville, NY, USA). Results were presented as geometric mean and standard deviation (SD) of gray levels.

### 2.6. Evaluation of Ocular Surface Inflammation

To explore the mechanism by which ectoine protects the ocular surface from damage in mice exposed to DS, the expression of pro-inflammatory cytokines TNF-α, IL-1β, and IL-6 and chemokines CCL3 and CXCL11 in corneas and conjunctivas at mRNA and protein levels was investigated in the murine dry eye model via a quantitative RT-PCR and immunofluorescence staining.

### 2.7. RNA Isolation, Reverse Transcription (RT), and Quantitative Real-Time PCR (qPCR)

Total RNA was isolated from the corneal epithelium and conjunctival tissues that were collected and pooled from 2 eyes (right and left) from untreated control mice, DS mice topically treated with ectoine at different concentrations (0.5, 1.0, and 2.0%), or PBS vehicle. Briefly, the RNeasy Plus Mini Kit (Qiagen, Valencia, CA, USA) was used for total RNA extraction according to the manufacturer’s instructions; RNA samples were quantified with a spectrophotometer (NanoDrop ND-1000; Thermo Scientific, Wilmington, DE, USA) and stored at −80 °C. The Ready-To-Go You-Prime First-Strand Beads (GE Heathcare, Piscataway, NJ, USA) were used to synthesize the first strand cDNA by RT from 2.0 µg total RNA, using previously described protocols [25].

Quantitative real-time PCR was performed in a reaction volume of 10 µL containing 4.5 µL cDNA, 0.5 µL TaqMan gene expression assay, and 5.0 µL TaqMan gene expression master mix using the QuantStudio 3 Real-Time PCR System (Applied Biosystems, Foster City, CA, USA). The thermocycler parameters were set at 50 °C for 2 min and 95 °C for 10 min, followed by 35 cycles of 95 °C for 15 sec and 60 °C for 1 min. The following TaqMan gene expression assays were used: GAPDH (Mm99999915_g1), TNF-α (Mm00443258_m1), IL-1β (Mm00434228_m1), IL-6 (Mm00446190_m1), CCL3 (Mm00441258_m1), and CXCL11 (Mm00444662_m1). A non-template control was included to assess DNA contamination. Results were analyzed via the comparative threshold cycle (Ct) method and normalized by GAPDH as an internal control.

### 2.8. Immunofluorescent Staining and Laser Scanning Confocal Microscopy

The mice eyes with lids in each group were excised, embedded in OCT compound (VWR, Suwanee, GA, USA), and flash-frozen in liquid nitrogen. Sagittal 8 µm cryosections from mouse globes were cut using a cryostat (HM 500; Micron, Waldorf, Germany) and stored at –80 °C until use. Then, immunofluorescent staining was performed as previously described [26]. In brief, the cryosections were fixed with cold acetone at −30 °C for 3 min. After blocking with 20% normal goat serum in PBS for 60 min, the samples were incubated with primary antibodies overnight at 4 °C. The primary antibodies were rabbit anti-mouse TNF-α and IL-1β (Santa Cruz Biotechnology, Dallas, TX, USA), and IL-6, CCL3, or CXCL11 (CUSABIO, Houston, TX, USA). The goat anti-rabbit secondary antibody Alexa-Fluor 488 (1:100) was used with DAPI for nuclear counterstaining. Digital images were captured at a wavelength of 400–750 nm and one μm z-step using a laser scanning confocal microscope (Nikon A1 RMP, Melville, NY, USA) with NIS Elements software version 4.20 (Nikon, Garden City, NY, USA).

### 2.9. Statistical Analysis

The Student’s *t*-test or Mann–Whitney U test was used to make comparisons between 2 groups. A one-way ANOVA test was used to make comparisons between three or more groups, followed by Dunnett’s post hoc test. *p* < 0.05 was considered statistically significant. The software GraphPad Prism (Version 9, GraphPad Software, Boston, MA, USA) was used for the statistical analysis.

## 3. Results

### 3.1. Efficacy of Ectoine at Protecting Corneal Epithelium in the Murine Experimental Dry Eye Model

#### 3.1.1. Corneal Smoothness

**Figure 1:**
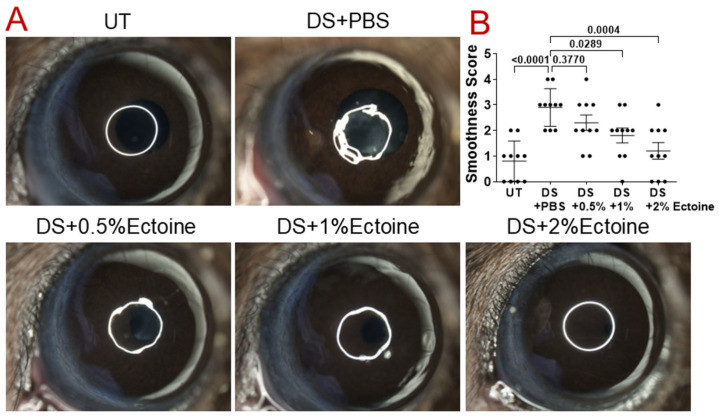
Corneal smoothness score evaluated via reflected white light rings in the murine dry eye model. (**A**) Representative digital images of the corneal surface were used to score corneal smoothness in UT mice, and DS mice treated with vehicle (DS + PBS) or ectoine at three concentrations (DS + 0.5%Ectoine, DS + 1.0%Ectoine, and DS + 2.0%Ectoine). (**B**) Quantitative corneal smoothness score results were summarized as mean ± SD from 10 eyes of each group of mice. Each dot represents one eye. *p* values are shown in the graph as compared with DS + PBS. DS, desiccating stress; Ect, ectoine; PBS, phosphate buffered saline; UT, untreated.

The regularity of a white light ring reflecting off the mouse corneas was used to evaluate their smoothness [24]. Compared with untreated (UT) normal mice (0.80 ± 0.78, Mean ± SD), the reflected white rings on the corneal surfaces of DS mice were largely distorted, with smoothness scores 2.90 ± 0.74, indicating damaged corneal smoothness and regularity. Topical administration of 0.5, 1.0, and 2.0% of ectoine in DS mice improved corneal smoothness in a concentration-dependent manner, and ectoine at 1.0 and 2.0% significantly restored the corneal smoothness to near or completely normal condition, with a corneal smoothness score of 1.80 ± 0.92 and 1.20 ± 1.03, respectively, as shown by the reflected white rings (Figure 1A) and the quantitative corneal smoothness scores from all groups of mice (Figure 1B).

#### 3.1.2. OGD Fluorescent Staining

**Figure 2:**
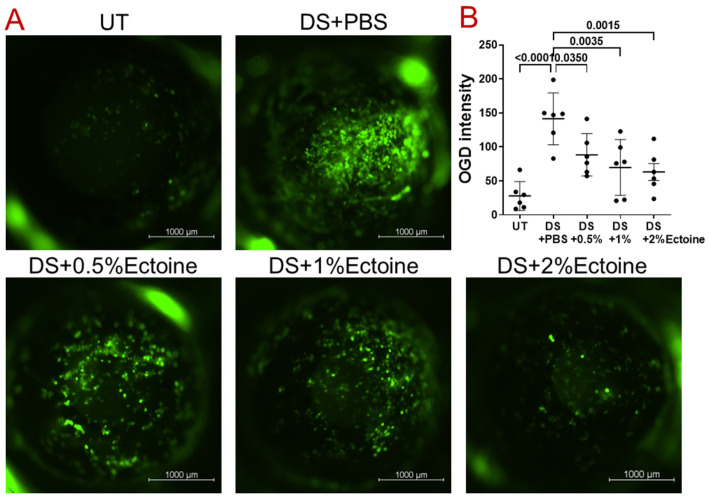
Corneal epithelial defect and permeability evaluated via Oregon Green dextran (OGD) fluorescent staining in the murine dry eye model. (**A**) Representative images of corneas stained with 1.0 µL of 50 mg/mL OGD in five groups of mice (UT, DS + PBS, and DS + 0.5%Ectoine, DS + 1.0%Ectoine, and DS + 2.0%Ectoine). (**B**) The mean fluorescence intensity in the central cornea with a diameter of 2 mm was evaluated after background correction of OGD staining in five groups of mice. Each dot represents one eye. Data are shown as Mean ± SD, *n* = 6; *p* values are shown in the graph as compared with DS + PBS.

OGD, a large molecular weight fluorescein, can be used to detect corneal epithelial defects and permeability [24]. Compared with the UT group, corneal uptake of OGD significantly increased with punctate and confluent dye staining in DS mice treated with vehicle (Figure 2A), mimicking human keratoconjunctivitis sicca, of which a hallmark is increased corneal epithelial permeability to the diagnostic dye sodium fluorescein. Treatment with ectoine at 0.5, 1.0, and 2.0% had a protective effect in a concentration-dependent manner. In particular, corneal OGD uptake appeared dramatically reduced in DS mice treated with 1.0 and 2.0% ectoine.

Quantitation of OGD fluorescence intensity showed similar patterns in corneal permeability. The OGD intensity was significantly higher (141.30 ± 38.17) in DS mice treated with vehicle than UT controls (27.96 ± 21.23). Topical administration of ectoine at concentrations of 0.5, 1.0, and 2.0% reduced corneal fluorescence intensity significantly to 88.30 ± 31.12 (*p* = 0.035), 69.83 ± 41.07 (*p* = 0.0035), and 63.19 ± 30.55 (*p* = 0.0015), respectively (Figure 2B).

All measures of disease pathology suggest that corneal epithelial defects are elevated in DS mice, while topical administration of ectoine protected the corneal epithelium from damage. In particular, the treatment with 2% ectoine appeared to have the strongest effect in this murine model of experimental dry eye. Thus, the mice group that received 2% ectoine were used for the following experiments.

### 3.2. Effect of Ectoine on Suppressing Pro-Inflammatory Cytokines in the Murine Experimental Dry Eye Model

Inflammation is a major driver of pathogenesis and may also contribute to corneal defects in dry eye disease [6]. To explore the effect of ectoine on inhibiting inflammation induced by desiccating stress, the expression of pro-inflammatory cytokines TNF-α, IL-1β, and IL-6 was investigated in this murine model of dry eye disease. 

**Figure 3:**
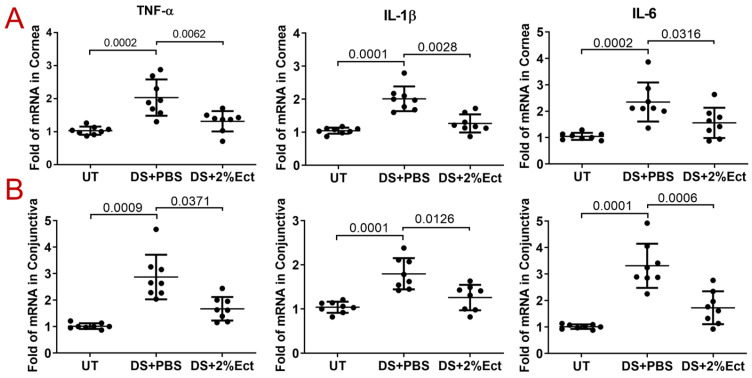
The expression of pro-inflammatory cytokines TNF-α, IL-1β, and IL-6 in the corneas and conjunctivas of dry eye mice. (**A**) The mRNA expression of TNF-α, IL-1β, and IL-6 increased markedly in the corneas of DS + PBS mice but was significantly suppressed by topical administration of ectoine (DS + 2%Ectoine). (**B**) The mRNA levels of TNF-α, IL-1β, and IL-6 were largely upregulated in the conjunctivas of DS + PBS mice, but significantly reduced in DS + 2%Ectoine mice. Data are shown as Mean ± SD; each dot represents one mouse, *n* = 8/group. *p* values are shown in the graph as compared with DS + PBS.

Compared with UT mice, the mRNA expression of TNF-α, IL-1β, and IL-6 was increased to 2.78 ± 0.59 fold (*p* = 0.0002), 2.01 ± 0.37 fold (*p* = 0.0001), and 2.3 ± 0.74 fold (*p* = 0.0002), respectively, in the corneal epithelia of DS mice; while topical administration of 2% ectoine significantly inhibited the mRNA levels of these three cytokines to near normal levels (1.57 ± 0.49 (*p* = 0.0062), 1.26 ± 0.27 (*p* = 0.0028), and 1.55 ± 0.57 (*p* = 0.0316)) (Figure 3A). Similar to corneal epithelia, the mRNA expression of TNF-α, IL-1β, and IL-6 in the conjunctiva of DS mice increased to 2.87 ± 0.84 fold (*p* = 0.0009), 1.80 ± 0.35 fold (*p* = 0.0001), and 3.31 ± 0.83 fold (*p* = 0.0001), respectively, but significantly decreased to normal or near normal levels (1.67 ± 0.44 (*p* = 0.0371), 1.25 ± 0.28 (*p* = 0.0126), and 1.72 ± 0.61 (*p* = 0.0006)) in DS mice treated with 2% ectoine eye drops (Figure 3B).

**Figure 4:**
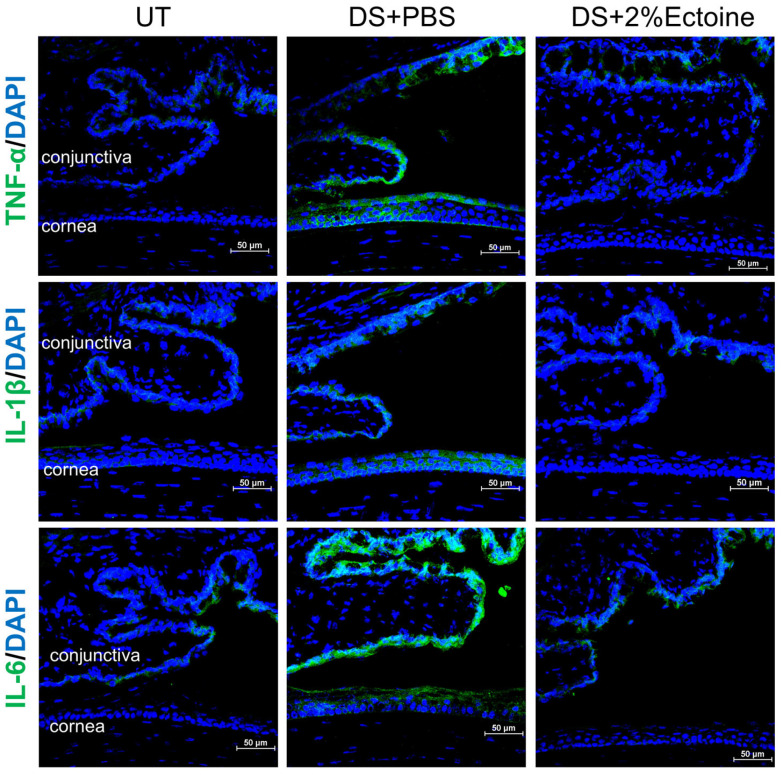
Protein production of TNF-α, IL-1β, and IL-6 in the corneas and conjunctivas of dry eye mice. Representative images of immunofluorescent staining show that the stimulated production of three pro-inflammatory cytokines in the corneas and conjunctivas of DS + PBS mice was largely suppressed in DS + 2%Ectoine mice. Scale bars, 50 µm.

Immunofluorescent staining of TNF-α, IL-1β, and IL-6 showed a consistent expression pattern at protein levels compared to mRNA levels in UT and DS mice treated with 2% ectoine or vehicle. As shown in Figure 4, these three cytokines were weakly expressed by some conjunctival cells but barely detected on corneas in UT mice. The desiccating stress markedly increased the immunoreactivity of these cytokines in both corneal and conjunctival cells. Topical eye drops of 2% ectoine largely reduced the immunoreactivity of these cytokines to levels that were similar to normal UT mice.

### 3.3. Effect of Ectoine on Suppressing Chemokines That Attract the Infiltration of Immune Cells to the Ocular Surface

The infiltration of immune cells including neutrophils, macrophages, dendritic, and T cells participates in the ocular surface inflammation of dry eye disease [27]. In addition to pro-inflammatory cytokines such as TNF-α and IL-1β, chemokines also play an important role in recruiting these immune cells to the inflammatory site on the ocular surface. Here, we evaluated the expression of the C-C motif chemokine CCL3 and CXC motif chemokine ligand CXCL11 in our dry eye model. 

**Figure 5:**
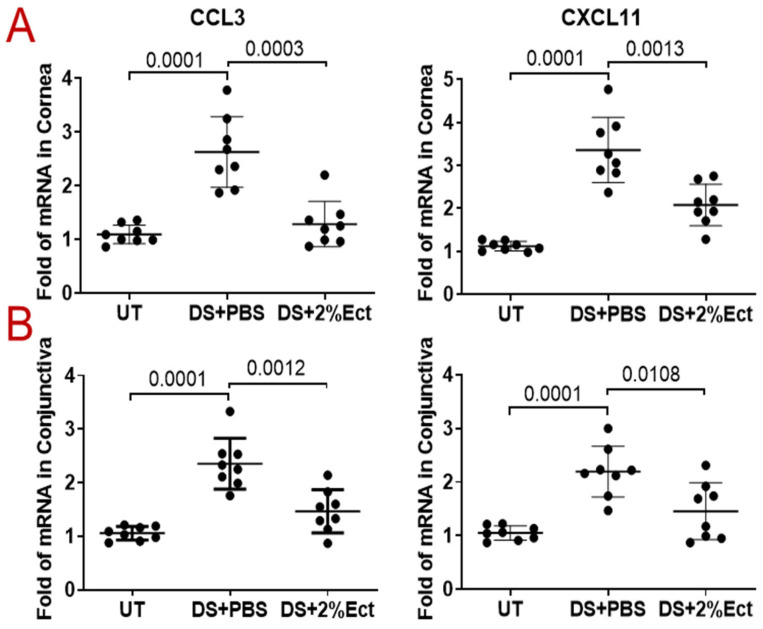
Compared with UT control mice, the mRNA expression of CCL3 and CXCL11 increased dramatically in the corneas (**A**) and conjunctivas (**B**) of DS + PBS mice but was suppressed significantly by topical ectoine administration (DS + 2%Ectoine). Data are shown as Mean ± SD; each dot represents one mouse, *n* = 8/group.

Compared with UT mice, the mRNA expression of CCL3 and CXCL11 was upregulated to 2.63 ± 0.66 fold (*p* = 0.0001) and 3.36 ± 0.76 fold (*p* = 0.0001), respectively, in the corneal epithelia of DS mice, while topical administration of 2% ectoine significantly inhibited the mRNA expression of these chemokines to near normal levels, 1.29 ± 0.42 (*p* = 0.0003) and 2.08 ± 0.48 (*p* = 0.0013) (Figure 5A). Like corneal epithelia, the mRNA expression of CCL3 and CXCL11 in the conjunctivas of DS mice also significantly increased to 2.36 ± 0.47 fold (*p* = 0.0001) and 2.19 ± 0.47 fold (*p* = 0.0001), respectively, but reduced to near normal levels, 1.47 ± 0.40 (*p* = 0.0012) and 1.45 ± 0.53 (*p* = 0.0108), respectively, with treatment with 2.0% ectoine eye drops (Figure 5B).

**Figure 6:**
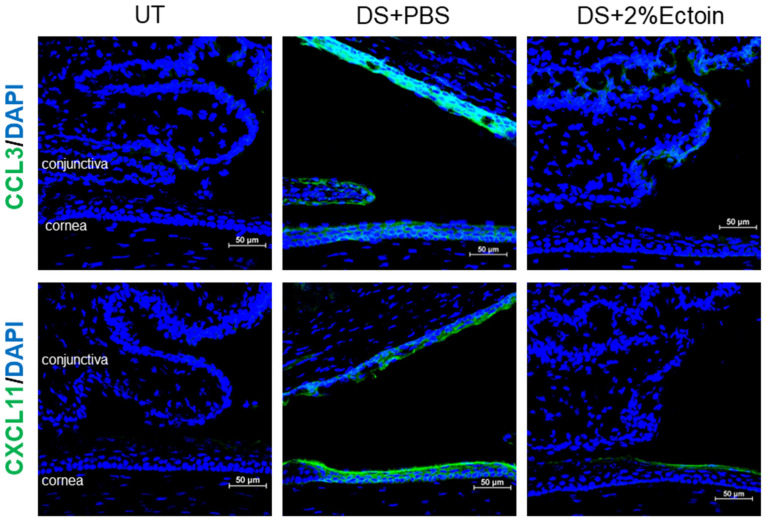
Protein production of CCL3 and CXCL11 in the corneas and conjunctivas of dry eye mice. Representative images of immunofluorescent staining show that the stimulated production of these two chemokines in the corneas and conjunctivas of DS + PBS mice was largely inhibited in DS + 2%Ectoine mice. Scale bars, 50 µm.

Immunofluorescent staining of CCL3 and CXCL11 showed a consistent expression pattern at the protein levels compared to mRNA levels in UT and DS mice treated with 2% ectoine or vehicle. As shown in Figure 6, CCL3 and CXCL11 staining was negative in corneal and conjunctival cells from UT mice, whereas desiccating stress markedly increased the immunoreactivity of these chemokines in both corneal and conjunctival cells. It appeared that CCL3 immunostaining was stronger in conjunctivas, while CXCL11 reactivity was stronger in corneas. Topical eye drops of 2% ectoine largely reduced the immunoreactivity of CCL3 and CXCL11 to very weak levels in the conjunctiva or cornea, respectively.

## 4. Discussion

Bacteria inhabit natural and artificial environments with diverse and fluctuating osmolalities, salinities, and temperatures. Many maintain cytoplasmic hydration, growth, and survival most effectively by accumulating compatible solutes when medium osmolality is high or temperature is low [28]. Ectoine, a cyclic tetrahydropyrimidine organic osmolyte, is a prominent member of stress protectants that is produced via an evolutionarily conserved biosynthetic pathway and was initially discovered in the halophilic bacterium *Ectothiorhodospira halochloris* [29].

Ectoine is a natural cell-protective and inflammatory-reducing molecule with an excellent tolerability and safety profile [14,30,31]. Ectoine has a strong binding capacity to water molecules and can form a protective hydration shield around biomolecules [32]. Ectoine can also promote the functionality of key cellular processes under unfavorable intracellular conditions [30]. Recent studies have suggested that ectoine can relieve the symptoms of different inflammatory diseases without serious side effects, such as lung inflammation, atopic dermatitis, allergic rhinoconjunctivitis [17,18], and dry eye disease [20,33]. However, the clinical trials are limited and there are no reports of animal studies investigating the role and molecular mechanism of ectoine in dry eye disease.

To evaluate whether the protective role of ectoine from bacteria could be utilized as a potential therapeutic treatment for human dry eye disease, the present study investigated the effect of the ocular surface protection and anti-inflammation of ectoine in a murine experimental dry eye model. Patients with dry eye often suffer eye discomfort including irritation, itching, burning, blurring, and photosensitivity. These symptoms are mainly derived from ocular surface epithelial defect due to chronic desiccation. We evaluated corneal epithelial cell pathology by measuring corneal smoothness and OGD fluorescent staining in our murine dry eye model.

Our experimental dry eye model has been used to investigate dry eye disease for two decades [22]. Consistent with previous reports [24], we observed that when compared with untreated normal mice, the reflected white light rings on the corneal surface were largely distorted, with significantly elevated smoothness scores in dry eye mice exposed to desiccation with PBS vehicle, indicating decreased corneal smoothness due to corneal epithelial defects. Topical ectoine administration in DS mice improved corneal smoothness with reduced irregularity in a concentration-dependent manner, and ectoine at 2.0% appeared to be an optimal dose that best restored the corneal smoothness to near normal condition, as shown by complete white rings and the lowest quantitative corneal smoothness scores (Figure 1).

Although corneal epithelial defects are usually examined via staining with 0.1% liquid sodium fluorescein clinically, OGD, a large size of 70 kDa fluorescein, can better detect corneal epithelial defects and permeability in a murine dry eye model [24]. Compared with the UT group, corneal uptake of OGD significantly increased with punctate and confluent dye staining in DS mice with PBS vehicle (Figure 2A), mimicking human dry eye, of which a hallmark is the increased corneal epithelial permeability to the diagnostic dye sodium fluorescein [34]. The quantitative fluorescein intensity of OGD staining confirmed the similar patterns to corneal uptake of OGD. Treatment with ectoine at 0.5, 1.0, and 2.0% had a protective effect in a concentration-dependent manner. The corneal OGD uptake was mostly significantly reduced to normal in DS mice treated with 2.0% ectoine eye drops.

Inflammation is a major driver of pathogenesis in dry eye disease, of which hyperosmolarity is a major etiological factor [8]. As an osmoprotectant, it is not clear how ectoine plays a protective role on ocular surface epithelia via its strong effect of preferential hydration. To explore the potential mechanism by which ectoine improves ocular surface epithelial disease in DS mice exposed to desiccating stress, we investigated the expression of pro-inflammatory cytokines and chemokines that induce ocular surface inflammation.

TNF-α, IL-1β, and IL-6 are major markers of ocular surface inflammation in dry eye. The expressions of these pro-inflammatory cytokines are largely stimulated in the tear and ocular surface of patients with keratoconjunctivitis sicca [35], as well as in murine models of experimental dry eye [36]. We observed that the mRNA levels of TNF-α, IL-1β, and IL-6 were significantly upregulated in the corneal and conjunctival epithelia of DS mice when compared with UT mice, while topical application of 2% ectoine significantly inhibited their mRNA expression to near normal levels (Figure 3). Immunofluorescent staining confirmed the expression pattern of TNF-α, IL-1β, and IL-6 at protein levels in UT and DS mice treated with 2% ectoine or vehicle (Figure 4).

Dry eye disease is also an immune-mediated ocular surface disorder. A variety of immune cells including neutrophils, macrophages, dendritic cells, and T cells are often infiltrated into the ocular surface, which enhances the inflammatory responses in dry eye disease [37,38]. Chemokines are known to play an important role in recruiting these immune cells into the inflammation site of the ocular surface. For example, the expression of CCL3 (macrophage inflammatory protein)-1α and CCL4/MIP-1β, and CXCL9, -10, and -11 have been found to increase in the tear film and ocular surface of patients with dry eye syndrome, especially in those with Sjögren’s syndrome, and the expression of CCL3/4 and CXCL11 at mRNA and protein levels was significantly correlated with the severity of the dry eye condition [27,39].

CCL3, mainly derived from neutrophils, is essential for the rapid recruitment of dendritic cells, and macrophage-derived CXCL11 is chemotactic for activated T cells. The expression levels of CCL3 and CXCL11 represent the potential infiltration of neutrophils and macrophages. Compared with UT mice, the mRNA expression of CCL3 and CXCL11 was significantly upregulated in the corneal and conjunctival epithelia of DS mice, while it was significantly inhibited to near normal levels by 2% ectoine eye drops. These patterns were also observed at the protein level via immunofluorescent staining.

Limitations and future studies: This study was the first attempt to explore the protective effects of ectoine on dry eye using a murine model. The study scope is limited in terms of elucidating the mechanisms of controlling inflammation in dry eye using ectoine. Many questions are remaining. Is it, in fact, impacting tear osmolarity? The answer is not clear because we did not directly measure the ocular osmolarity. An important clinical study has reported that changes in tear osmolarity do not correlate significantly with changes in patient symptoms or corneal fluorescein staining in dry eye disease [40]. We hypothesize that the protective effects of ectoine, an amino acid derivative, might contribute to its unique effect of strong preferential hydration [14], but not to its function as a lubricant. To clarify how this works, further studies are required to investigate the cellular pathway and molecular mechanism, such as Th1 and Th17 responses, which are well established as playing an important role in the pathogenesis of dry eye disease [41,42].

## 5. Conclusions

In conclusion, corneal epithelial defects and inflammation were detected in a mouse model of dry eye disease, while topical application of ectoine inhibited these pathological changes in a concentration-dependent manner. Ectoine at 2% appeared to be an optimal concentration that significantly reduced measurements of ocular surface epithelial disease and suppressed the increased expression of pro-inflammatory cytokines and chemokines in the dry eye mouse model.

These findings demonstrate that ectoine is able to significantly reduce the hallmark pathologies associated with dry eye disease in a well-established mouse model and therefore may be a promising candidate for the treatment of human disease. Additional studies are necessary to further explore the molecular mechanisms of the action of ectoine, as well as evaluate whether these observations may translate to the development of new therapeutic options for the potential treatment of dry eye disease in humans.

## Data Availability

The data presented in this study are available in this article.
